# Towards 3D crystal orientation reconstruction using automated crystal orientation mapping transmission electron microscopy (ACOM-TEM)

**DOI:** 10.3762/bjnano.9.56

**Published:** 2018-02-15

**Authors:** Aaron Kobler, Christian Kübel

**Affiliations:** 1 Karlsruhe, Germany, private contribution; 2 Institute of Nanotechnology (INT), Karlsruhe Institute of Technology (KIT), Hermann-von-Helmholtz-Platz 1, 76344 Eggenstein-Leopoldshafen, Germany; 3 Karlsruhe Nano Micro Facility (KNMF), Karlsruhe Institute of Technology (KIT), Hermann-von-Helmholtz-Platz 1, 76344 Eggenstein-Leopoldshafen, Germany

**Keywords:** ACOM-TEM, 3D reconstruction, in situ testing, quantitative crystallographic analysis, STEM

## Abstract

To relate the internal structure of a volume (crystallite and phase boundaries) to properties (electrical, magnetic, mechanical, thermal), a full 3D reconstruction in combination with in situ testing is desirable. In situ testing allows the crystallographic changes in a material to be followed by tracking and comparing the individual crystals and phases. Standard transmission electron microscopy (TEM) delivers a projection image through the 3D volume of an electron-transparent TEM sample lamella. Only with the help of a dedicated TEM tomography sample holder is an accurate 3D reconstruction of the TEM lamella currently possible. 2D crystal orientation mapping has become a standard method for crystal orientation and phase determination while 3D crystal orientation mapping have been reported only a few times. The combination of in situ testing with 3D crystal orientation mapping remains a challenge in terms of stability and accuracy. Here, we outline a method to 3D reconstruct the crystal orientation from a superimposed diffraction pattern of overlapping crystals without sample tilt. Avoiding the typically required tilt series for 3D reconstruction enables not only faster in situ tests but also opens the possibility for more stable and more accurate in situ mechanical testing. The approach laid out here should serve as an inspiration for further research and does not make a claim to be complete.

## Findings

For the study of nanostructured material with feature sizes <100 nm, transmission electron microscopy (TEM) is the method of choice due to its high spatial resolution [1–4]. Even though the electron-transparent TEM specimen lamellas are often only a few tens of nanometers thick, overlapping structures, such as crystallites of different orientations and phases, are unavoidable and typically unwanted. Nevertheless, in some cases it is even desirable to have overlapping crystallites to reduce the influence of the free surface and to increase the interaction between crystallites to represent bulk behavior, especially related to in situ studies (e.g., tensile or thermal testing) inside the TEM [2,5–9]. However, the 2D projection of a 3D volume with overlapping structures results in an incomplete picture, which can lead to misinterpretation. Hence, a full 3D reconstruction is desirable to relate the internal structure of a volume (crystallite and phase boundaries) to properties (electrical, magnetic, mechanical, thermal). 3D reconstructions using X-ray diffraction (XRD) [10–13], electron back scatter diffraction (EBSD) [14–17] and TEM have been presented [18–21]. However, only TEM has the spatial resolution to resolve the smallest structures of nanocrystalline material [1–4,15,22–23]. While 3D-EBSD uses volume slicing and imaging [15–17,24], XRD- and TEM-tomography require a sample tilt series for the 3D reconstruction [10–11,20,25–26].

Crystal orientation mapping has become a standard method for crystal orientation and phase determination. However, 3D crystal orientation mapping remains challenging and has been reported only a few times [18–19]. 2D crystal orientation can be mapped using three methods inside the TEM: Kikuchi diffraction, diffraction pattern reconstruction from conical dark field images and spot Bragg diffraction [22–23]. Kikuchi diffraction was the first to be used in the TEM [22–23]. However, it became popular in the scanning electron microscope as EBSD, as the samples do not have to be electron transparent and provide enough interaction volume to reveal strong Kikuchi lines [22–23]. The thinner the specimen lamella the weaker the Kikuchi signal, which leads to a contradiction of spatial resolution and quality of crystal orientation data for the TEM. Conical dark field imaging (CDFI) was used in the 3D-orientation mapping in transmission electron microscope (3D-OMiTEM) method to first reconstruct the diffraction pattern and then the 3D crystal structure [19]. Spot Bragg diffraction, in combination with template matching for the creation of crystal orientation maps, mostly referred to as automated crystal orientation mapping transmission electron microscopy (ACOM-TEM), has become the most prominent method [22–23,27–32]. The template matching, which cross-correlates the experimental diffraction pattern with a database of simulated diffraction patterns covering all crystal orientations and phases for the investigated material, is a fast and robust evaluation routine. Moreover, ACOM-TEM is a quantitative method with respect to sample parameters like grain size, twin density, and orientation density [28–29,33]. The quantitative analysis capability makes it a very unique tool for the investigation of nanomaterials. However, it has been shown that the orientation analysis is challenging when crystals partially overlap in projection, and the orientation of the main crystal contributing to the diffraction pattern must be identified [34]. This has left some uncertainty in the interpretation of 2D orientation maps.

The combination of in situ testing with 3D crystal orientation mapping remains a challenge in terms of stability and accuracy. Avoiding sample tilting for the 3D reconstruction would enable much faster mapping of nanomaterials in situ inside the TEM. Further, more stable and more accurate in situ mechanical testing would be possible.

In the following, a pathway towards 3D reconstruction from 2D crystal orientation maps is described and first results presented.

The starting point for the 3D reconstruction is an ACOM map containing experimental spot diffraction patterns originating from a Pd thin film deposited by radio frequency (RF) magnetron sputtering. The orientation map was acquired on a Philips Tecnai F20 ST TEM instrument which was equipped with a NanoMegas ASTAR system in combination with a Topspin (Appfive) software. A detailed description of the sample preparation and data acquisition can be found in [34]. For these preliminary results, only a few pixels of the whole crystal orientation map were analyzed as the analysis is not integrated into the template matching program ASTAR (NanoMegas) and had to be performed manually.

**Figure 1:**
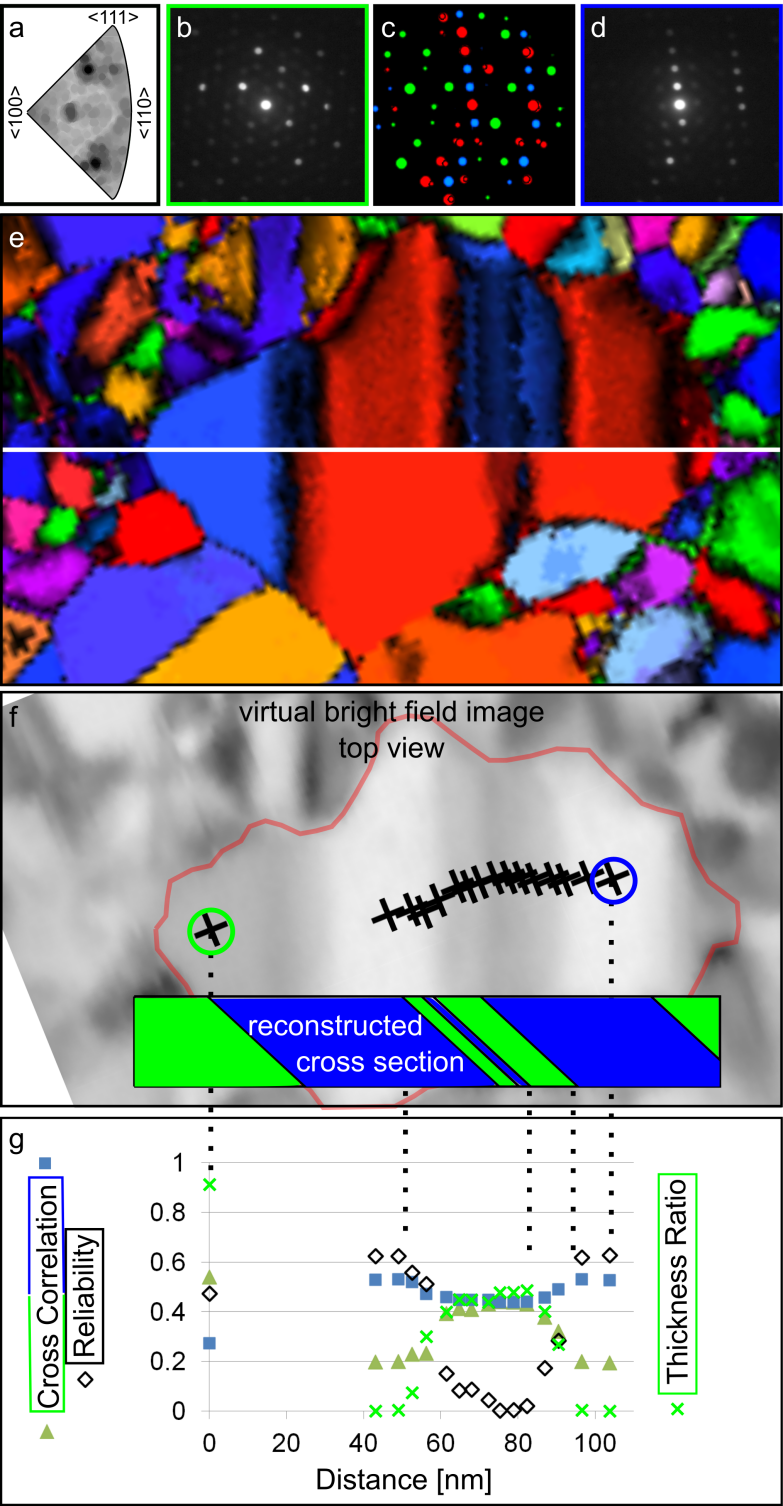
(a) Inverse pole figure (for fcc) with cross-correlation indices of a simulated and experimental diffraction pattern as gray values. The darkest spot has the greatest cross correlation index and determines the best match, and hence determines the orientation of one pixel from an orientation map as shown in (e). (b,d) Spot diffraction pattern of two unique crystal orientations. Positions where (b) and (d) originate from are highlighted in (f) with green and blue circles, respectively. (c) Overlay of both orientations (b green) and (d blue) (red indicates the shared diffraction spots of both orientations). (e) Two halves of an orientation map belonging to the same area and processed with two filter approaches (color code: crystal orientation). (f) Virtual bright field map that highlights one grain with two twin crystallites and the evaluated positions. The reconstructed cross section (based on the evaluation in (g)) is overlaid. (g) Cross-correlation indices for both orientations (b,d), where reliability and thickness ratio of the (b)-orientation are plotted versus the position of the markers.

Using template matching, the orientation of each pixel is determined by the best match, which is the highest cross-correlation index between the simulated diffraction pattern of all crystal orientations (and phases) and the experimental spot diffraction pattern [32]. In the case of overlapping crystallites, the experimental diffraction pattern is a superimposed result of the corresponding individual crystal orientations [34–35]. Hence, superimposed diffraction patterns have more than one match with a cross-correlation index above a given threshold. This can be visualized in an inverse pole figure containing the cross-correlation indices of all simulated orientations with one experimental diffraction pattern (Figure 1a, dark areas indicate high cross-correlation indices). It has been shown that the template matching result of the superimposed diffraction pattern is the same for 0° and 180° sample orientation [36]. This suggests that dynamic scattering has only a small negligible influence on the template matching result.

In the following, a few pixels from a grain with two twin crystallites (taken from an orientation map) were analyzed to calculate the orientation overlap in projection from the superimposed diffraction pattern. Experimental diffraction patterns of the two twin crystallites are shown in Figure 1b,d. A superimposed diffraction pattern with varying intensity contributions is detected at positions where both twin crystallites overlap in projection. In Figure 1c the matched simulated diffraction pattern of both twin crystallites are overlaid and their unique diffraction spots are highlighted in the corresponding colors. Once the unique diffraction spots from the contributing crystal orientations are identified [34–35], defined as the reduced simulated diffraction pattern (RSDP), they can be used as masks to calculate their mean intensities (gray values) (mI*_i_*). Here the values of mI*_i_* are background corrected, which is interpolated from the space between the diffraction spots. For simplicity, this data evaluation was all performed in Adobe Photoshop.

Based on the mean intensities, the thickness ratio of the contributing crystal orientation in projection is derived as follows.

We use the simplified assumption of linear scattering, neglecting dynamic effects:

[1]
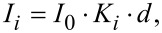


where *d* is the material thickness, *K_i_* is a scattering strength, *I*_0_ is the intensity of the primary beam and *I_i_* is the intensity of the beam after traversing a crystal.

We define a theoretical scattering ratio (SR) of the intensities from two crystal orientations with the same thickness *d*:

[2]
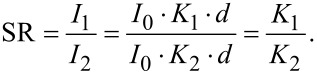


The diffraction intensities *I_i_* should be calculated using a proper kinematic diffraction simulation. Here, we approximate the scattering strength *K_i_* of the contributing crystal orientations by the mean area (mA*_i_*) of the RSDP disks (area of disks divided by the number of disks), which is derived from template matching (ASTAR).

For overlapping crystallites with unknown thicknesses *d_i_* and the local thickness *d*_tot_ in projection the intensity ratio rI is:

[3]
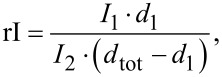


which is equivalent to the experimental intensity ratio of the mean intensities mI*_i_*. Considering the aforementioned approximation, this results in the thickness *d_i_*:

[4]
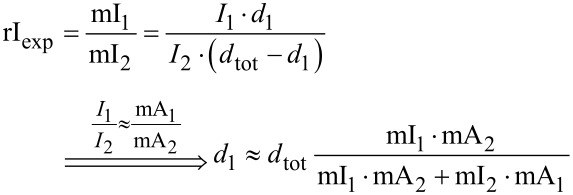


This thickness calculation was done for all markers of Figure 1f setting the total thickness *d*_tot_ to 1 to receive a relative ratio. Comparing the intensities of the diffracted beams with the primary beam, an absolute thickness could be calculated. Figure 1f is a virtual bright field (VBF) image reconstructed by placing a virtual aperture in the diffraction pattern. To compare the orientation contribution of each pixel to the cross-correlation indices for both contributing twin crystallites and to their reliability (ratio of cross-correlation indices for the tested orientations), all three measures are plotted versus the distance (Figure 1g). The distance is measured from the left green marker in Figure 1f to all others, normal to the twin boundary.

Although all three curves follow similar trends (apart from being flipped upside down, as blue cross-correlation index and reliability), it was shown that the cross-correlation indices and reliability values cannot be used as a measure for the thickness ratio [34]. To illustrate this, Figure 1e shows two halves of a crystal orientation map of the same area but evaluated with two different filter settings. The two twin crystallites of the grain, marked in Figure 1f, appear completely different dependent on the filter settings.

**Figure 2:**
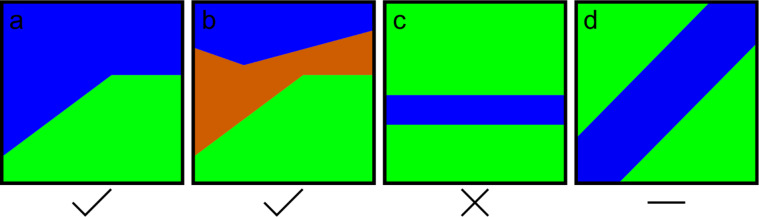
Illustrated cases of overlapping crystallites in cross section (different colors represent different crystal orientations).

Taking the thickness ratio as granted, the 3D reconstruction still misses one critical step. Figure 2 shows several cases of overlapping crystallites to illustrate the challenge. The thickness ratio of two crystallites overlaid in projection can directly be taken to reconstruct the 3D volume, apart from the mirror symmetry (upside down flip of Figure 2a). Three crystallites (Figure 2b) have *n*! permutations, where *n* is the number of overlapping crystallites. Nevertheless, the thickness ratio can be directly taken for the 3D reconstruction. A sandwich constellation of two twin crystallites as shown in Figure 2c is an unsolvable case for the 3D reconstruction from the thickness ratio. However, if the interface is slightly tilted as shown in Figure 2d, the boundary conditions as coincident site lattices (e.g., CSL Σ3, Σ9) support the 3D reconstruction. One could think of a brute force “3D puzzle” approach. An algorithm places certain crystal orientations in a random layer depth and compares it with a neighbor configuration and checks if the boundary condition of matching lattices is fulfilled. If not, it starts over with a different configuration.

Starting from 40 nm in the graph (Figure 1g) the thickness ratio is 0% green, increasing linearly until about 60 nm. The same applies for 95–80 nm, where the thickness ratio linearly increases. Based on the ratio plateau from 60–80 nm alone, there would be two overlapping twin crystallites (a case such as that in Figure 2a). However, the contributing crystallites are twins with defined, mainly straight and symmetric crystal plane relations. Deviations of a linear thickness ratio can result from multiple overlapping and inclined twins as presented in the inset of Figure 1f. The slope of the twin boundary was assumed from the thickness-ratio trend between 80 and 95 nm (Figure 1g).

What other hurdles need to be considered? The comparison of the intensity ratio from the superimposed experimental diffraction pattern with the area ratio of the corresponding simulated diffraction pattern (Equation 4) was the easiest approach, but not necessarily the best. Experimental intensities should be compared with simulated intensities and the areas are only an indirect measure of the intensities, which were easy to access with the NanoMegas software ASTAR. A full integration of the simulation would be possible, but was beyond the scope of this paper.

The assumptions of Equations 1–4 are based on linear mixing and dynamic scattering is neglected. Here, beam precession was used for data acquisition. It was shown that beam precession enhances the experimental diffraction pattern by suppressing the dynamical scattering [32,37–40]. Nevertheless, dynamic scattering adds up to the background and will influence the suggested data processing.

Certain (low zone axis) diffraction patterns from superimposed crystal orientations, Os, form a false pattern, Of (appearing as a high zone axis), which the template matching mistakes as a crystal orientation, which is non-existent in the mix [34]. The resulting false orientation, Of, of the experimental diffraction pattern has the highest cross-correlation index with its matched simulated diffraction pattern. Template matching itself seems to offer a solution to avoid the confusion: A diffraction pattern of lower-indexed orientations, Os, could be superimposed and compared with the best matched Of. If the superimposed orientations look like the false one (Of *≈* Os_1_* +* Os_2_), the false orientation could be filtered out.

## Conclusion

To summarize, the superimposed diffraction pattern from overlapping twin crystallites was used to reconstruct the 3D volume. The simple approach, laid out here, showed that the diffraction pattern contain the necessary information for the 3D reconstruction. This is in contrast to the Kikuchi pattern, which is determined mainly by the exit planes of the lattice, and hence does not provide enough information for a reconstruction [22,41–42]. Smart reconstruction algorithms are necessary and novel direct electron detection cameras in combination with advanced scanning optics can support the development towards 3D reconstruction from 2D projection images. This paper should inspire further research in this direction with the aim of using the 3D reconstruction in an in situ test series inside the TEM to receive a more complete picture of the material and to relate the material properties to structural changes.
